# Building an evidence base for stakeholder engagement

**DOI:** 10.1126/science.aat8429

**Published:** 2018-08-10

**Authors:** James V. Lavery

**Affiliations:** Members of the Havasupai tribe pray over blood samples at Arizona State University. Disregard for how the research could undermine the tribe’s interests led to a lawsuit and out-of-court settlement

Science is a social enterprise. Many scientific programs interact with a wide range of communities and stakeholders to secure various types of access and permission, to seek cooperation and collaboration for scientific studies, to fulfill regulatory and ethical requirements, and to try to shape research strategies and to improve the translation of their findings into policy or practice. But these interactions are motivated disproportionately by the interests and goals of the scientific programs and less by the need to elicit and understand their implications for stakeholders. However, there is increasing recognition that substantive community and stakeholder engagement (CSE) can improve the performance, and even make or break the success, of some science programs by providing a means of navigating, and responding to, the complex social, economic, cultural, and political settings in which science programs are conducted. For CSE to become more widely accepted by funders and researchers, and to contribute more conspicuously to the success of science programs and policy, it will have to establish a more coherent and convincing body of evidence about the nature of CSE strategies and their specific contributions to the performance of science programs.

The zeal that drives scientists in their quest for discovery and their deep-rooted faith in the scientific enterprise can sometimes lead them to underestimate, or disregard, the potential for their actions to negatively affect the interests of stakeholders beyond the immediate frame of reference of their scientific protocols. For example, Ashkenazi Jews faced stigmatization and discrimination on the basis of findings of population-genetics research (1), and unauthorized research on historical human migration patterns damaged the collective cultural identity of the Havasupai tribe of Arizona (2). Despite the importance of such harms, the dominant ethics paradigms in science–scientific integrity and human-subject research protections—provide little guidance about how to anticipate and avoid them.

A common intuition is that these harms can be mitigated by CSE. The idea has attracted interest in a wide range of disciplines, including sustainable development (3), regulation of new biotechnologies (4), and humanitarian emergencies (5), along with long-established practices in community-based participatory research (6), patient engagement in clinical research (7), and global health (8).

Yet, as one recent commentary about CSE noted, “there is limited empirical evidence on the best practices for stakeholder engagement and even less on evaluation of engagement demonstrating the association between the quality and quantity of engagement and research outcomes” (9). This lack of evidence about CSE could be the sustaining force for a self-fulfilling prophecy, because those with the authority to make budget decisions for science programs lack clarity about the circumstances under which CSE is necessary, its appropriate scope and form, and a clear and coherent value proposition for how CSE improves the ethics of research and enhances the impact of their investments. The result is often expressed as skepticism or indifference to the potential value of CSE.

## KEY CHALLENGES

First, the generation of useful and comparable evidence for CSE is complicated by the absence of an agreed theory of CSE. What are its constituent elements? What mechanisms are involved? What programmatic and ethical outcomes does it produce and under what circumstances? And how do these vary according to the nature of the science and the specific settings of application? Answers to these individual questions would not only provide insights about how CSE works in various contexts but would also facilitate the development of useful theory, which will be essential to move CSE beyond a static and critically unexamined set of practice conventions.

Second, the coherence and comparability of the evidence is undermined by the extraordinary degree of variability in the working language for CSE. Concepts, such as “engagement,” “sensitization,” “mobilization,” “empowerment,” and “trust-building,” are often conflated and interchanged casually, even though the goals and outcomes they imply differ substantially. Similarly, assumptions about what constitutes the relevant “community,” or who should be counted as a legitimate “stakeholder,” are often poorly stipulated or specified. This conceptual ambiguity and heterogeneity compounds the problem of insufficient precision and explicitness in the reporting of the social processes and outcomes associated with the prevalent concepts, which frustrates efforts to compare study findings.

Third, there is a tendency to think narrowly about CSE, or to emphasize or exaggerate some aspects relative to others. For example, CSE strategies tend to rely heavily on mechanisms such as community advisory boards, which typically provide limited and uncertain representation of the full range of relevant stakeholder interests and perspectives. They also emphasize communications and various strategies for developing and delivering “key messages” to educate host communities about the goals and merits of the science program. The provision of information is a necessary aspect of CSE but is often emphasized at the expense of listening to and acknowledging the interests of stakeholders. For example, a recent HIV pre-exposure prophylaxis trial for women was critically undermined when it was discovered that many of the participants were simply not using the study product (10). The women’s interests in the trial—their reasons for participating—were at odds with the researchers’ expectations, but these were not identified through the conventional engagement mechanisms.

Fourth, the circumstances described above exacerbate an emerging tension between moves to standardize CSE practices and measurement strategies in science programs (5, 11) and the need to customize them to account for unique social, economic, political, and cultural complexities that shape the contexts within which CSE strategies are executed. Even relatively simple CSE strategies involve multiple interacting components; long, nonlinear implementation chains; and complex sets of human interests, relationships, and associations. In many cases, the motivating interests, reasoning, and behavior of stakeholders are active mechanisms in the performance of the science program itself (12). The development of generic approaches to CSE in the form of core principles (4), best practices (5), and key metrics and indicators (11) provides useful momentum for the development of CSE as a legitimate domain of knowledge generation but is unlikely to provide reliable guidance precisely when effective CSE might be most valuable, that is, when the science is controversial or when the human contexts are most complex and/or contentious. Undue confidence in standardized approaches to CSE could inadvertently weaken the force of appeals for better evidence.

## IS EVIDENCE WORTH THE INVESTMENT?

In light of these challenges, the broader question for science policy and programming is whether funders should accept that CSE has sufficient potential value for the performance of certain science programs—in addition to the ethical rationales that likely motivate the majority of investments in CSE—to warrant greater investment in an appropriate evidence base. The uncertainty rests on some unresolved, but fundamental, questions about the relationship between science programs and those who might have legitimate grounds to be considered stakeholders. Under what circumstances is CSE necessary? How much and what kind of CSE is necessary? What standing do stakeholders have to assert their interests in any science program and by what processes? What factors should determine the weight that any set of stakeholder interests should carry? What specific obligations should the scientific program be required to acknowledge and accept, including obligations to make changes to their protocols or practices to avoid setting back—that is, harming—stakeholder interests? More broadly, how should science programs be planned, designed, and managed—including the necessary flexibility in budgets and protocols—to allow them to act on valuable stakeholder insights to improve their protocols, practices, and impact and to make changes to avoid harm? These questions have deep normative implications, but, without adequate empirical evidence, they are destined to remain marginal curiosities. Table S1 provides an overview of some of the key components of CSE strategies and a selection of some of the current gaps in evidence.

## DESIGN AND MANAGEMENT

Despite the linguistic and conceptual variability described above, CSE has a relatively stable core logic. Science programs are often designed, and final protocol and budget decisions made, remotely from the settings and populations in which they will be conducted. Although scientists tend to emphasize the potential benefits of their work, their programs can also feel like an imposition for some stakeholders and, in some cases, have negative implications for them. For example, comparative trials of new agricultural biotechnologies versus conventional crops can disrupt local market dynamics for small-holder farmers and create discord among neighbors. Therefore, some process is required to identify these potential implications and ensure that the appropriate understandings, agreements, and authorizations are sought from those whose interests may be at stake.

Analogous engagement challenges in other sectors—in particular, private-sector product and service design and supply-chain management—have fueled enormous investments, innovation, evidence generation, and industry-wide adoption of practices in customer-relationship management, customer-experience management, and a wide range of human-centered design strategies. These developments have resulted in paradigm shifts in the way the interests and insights of consumers are elicited and incorporated into product and service design and development processes. For example, human-centered design strategies have produced acclaimed products and services, from the first Apple computer mouse to award-winning educational programs to innovations in financial technology. These innovations share three main features. First, they are built on insights from consumers about their experiences with the products or services in question. Second, their development was motivated by the absence of appropriate management strategies that could reliably deliver consumer insights to inform product and service design. And third, they are highly transferable models, applicable wherever consumers’ reasoning and behavior play a role in the performance of the product or service.

The relevance and importance of these features for CSE cannot be overstated. Insights about stakeholder interests and perspectives provide the ethical foundation for CSE (13). But they also offer unique value as a means of critically examining, and refining, the design of science programs in response to the specific circumstances of a given research setting. As such, these insights about stakeholder interests also provide an important unit of analysis for a great deal of the necessary empirical research on CSE: What interests do stakeholders have in our science programs? How can we best address them? How do stakeholders want to be engaged? And how can engaging with stakeholders improve the performance of science programs?

Like customer-relationship management, or human-centered design, CSE involves the design and management of social processes. An early experience of the Eliminate Dengue program (now called the World Mosquito Program) (14) offers a useful illustration. In its initial open-release trials of Wolbachia-infected mosquitoes for population replacement in pursuit of a scalable dengue virus–transmission control strategy, Eliminate Dengue engaged a wide range of stakeholders in Queensland, Australia (14). The aims were to facilitate successful trials and to avoid imposing the technology on the population against its will. The CSE effort was widely considered to be successful. Though, for the reasons elaborated above, there was no established standard of “success” to guide an evaluation. Our case study of the Eliminate Dengue CSE strategy sought to critically examine the perceptions of success through in-depth interviews with a wide range of stakeholders, including Eliminate Dengue team members, regulators, a local federal cabinet minister, and local business owners and residents. On the basis of this study (14), I highlight six specific features that appear to have contributed to an impressively uniform perception of success among stakeholders: (i) consistent support (enabling conditions) from the funder and implementation partner to prioritize CSE activities, (ii) clear and consistent leadership to establish CSE as a key priority within the program, (iii) an inclusive view of stakeholders, (iv) a proactive approach to eliciting stakeholder interests and insights and a willingness to be flexible with the design and conduct of the trials in response to them, (v) a clear and coherent set of guiding principles and ethical commitments to stakeholders, and (vi) an explicit management strategy, effectively integrated with the general day-to-day program management, that operationalized the program’s guiding principles and ethical commitments and adopted stakeholder interests as a central focus of overall program management (14).

## WHAT CAN FUNDERS DO?

A number of major funders of science programs have already made substantial investments in research on CSE, including the Wellcome Trust, the National Institutes of Health, and the Bill & Melinda Gates Foundation. These investments have helped to illuminate some of the latent potential of CSE, but the limited evidence they have generated remains largely unknown among funders and generally insufficient to overturn a seemingly common view of CSE as simply another administrative requirement. Funders have a unique power to reframe this narrative, and better justify investments in evidence about CSE, by emphasizing its potential to improve the performance, as well as the ethics, of science programs.

In addition, although a central premise of CSE is that “feedback” from stakeholders is important, science programs are usually not structured in ways that permit meaningful revisions or refinements to programs—particularly to their protocols and budgets—in response to insights and feedback from stakeholders. Funders could substantially advance the mutual value of CSE for researchers and stakeholders by experimenting with more flexible and responsive management strategies, including innovations in protocol and budget processes, and studying the implications for various aspects of program performance. A timely example of this type of innovation comes from the Canadian International Development Research Centre (IDRC), which has recently published results of the implementation of a new tool it has developed to assess the quality of the research it funds, called Research Quality Plus (RQ+) (15). The development of the tool reflects IDRC’s acknowledgment of “the crucial role of stakeholders and users in determining whether research is salient and legitimate. It focuses attention on how well scientists position their research for use, given the mounting understanding that uptake and influence begins during the research process, not only afterwards” (15).

“…science programs are usually not structured in ways that permit meaningful revisions…in response to…stakeholders.”

In many scientific fields, a lack of agreement on nomenclature and conceptualization has presented obstacles to progress and has required extensive negotiations and deliberations, often in the form of specific conferences or consensus-building processes. In many cases, these initial deliberations have been critical for the advancement of the discipline and have given rise to some enduring governance structures, such as the International Union of Pure and Applied Chemistry and the *Diagnostic and Statistical Manual of Mental Disorders* of the American Psychiatric Association. For CSE, those funders who are most committed to building an appropriate evidence base might form a consortium to shape a working consensus on basic concepts and nomenclature for CSE to ensure that evidence is built on a sound conceptual architecture, before endorsing and adopting specific “standards” of practice for CSE.

At a minimum, funders should examine their current investments in CSE associated with science programs and ask whether these investments are contributing to the evidence base for CSE. Some of the necessary insights about how CSE works might be achieved simply by encouraging better reporting and scrutiny of the CSE strategies already being implemented in many science programs: What were the aims of these strategies? Did they work as expected? How and why did they work, or not work, in various contexts? What outcomes were attributable to the CSE? And how, if at all, were these outcomes conceptualized and measured? More production and reporting of this type of evidence should eventually reduce unproductive conceptual and linguistic variability and could provide valuable insights to improve theories of change for how CSE works and identify what tailoring and scaling might be required by different contexts. Improved reporting on these questions could also provide momentum for a broader research agenda for CSE, which could prove to be valuable across a wide range of scientific disciplines. Table S1 offers a point of departure for such a research agenda.

Because there is a self-evident sense in which stakeholders ought to have some say in what is done to them, with them, or on their behalf, funders have already incorporated CSE into many of their science programs. Perfect agreement about the best ways to conceptualize and design CSE strategies is not necessary to improve funding and implementation practices. An empirical evidence–based approach will eventually sort out how, and under what circumstances, CSE adds both ethical and practical value to science programs. A management-oriented approach to evidence generation that focuses on the interests and experiences of stakeholders may yield important insights about how CSE functions in various contexts, analogous to strategies used in customer-relationship and -experience management and human-centered design strategies in product and service development. Such an approach may offer critical insights about how the deeper ethical goals of CSE *(*13*)* might be more reliably accomplished. Research funders and implementation partners can play a critical role in enabling and establishing this evidence base to guide the appropriate utilization of CSE strategies in science programs.

## SUPPLEMENTARY materials

www.sciencemag.org/content/361/6402/554/suppl/DC1
