# Supplementary material for: Building an evidence base for stakeholder engagement
Source: Science. 2018 Aug 10;361(6402):554–6. doi: 10.1126/science.aat8429 (PMC7745122; doi:10.1126/science.aat8429)
Supplement: Supplementary file 1 [file SCIENCE-361-6402-554-s001.pdf]

www.sciencemag.org/content/361/6402/554/suppl/DC1

Supplementary Materials for

Building an evidence base for stakeholder engagement

James V. Lavery*

*Corresponding author. Email: jlavery@emory.edu

Published 10 August 2018, *Science* **361**, 554 (2018)
DOI: 10.1126/science.aat8429

This PDF file includes:

Table S1

Table S1: Gaps in evidence for community and stakeholder engagement

CSE Component	Selected examples of gaps in the CSE evidence
Foundations	
**Conceptual Foundations**	-how are key terms in CSE understood by funders, researchers and stakeholders<br>-what differences in CSE practices are associated with different terminology (e.g., “mobilization” vs. “engagement” vs. “empowerment”)
**Ethical Foundations**	-what value do funders, researchers and stakeholders ascribe to CSE?<br>-what ethical (and instrumental) goals do they associate with CSE?<br>-what ethical commitments do funders and science programs make to stakeholders and host communities?<br>-what obligations do funders and researchers believe they have to stakeholders?<br>-what obligations do stakeholders believe funders and researchers have to them?<br>-how do IRBs evaluate CSE strategies when human subjects are involved?
**Enabling Conditions**	-Under what circumstances do funders of science programs support CSE strategies for their investments?<br>-what assumptions inform budget requests and funding decisions for CSE?<br>-what costs are associated with CSE strategies?<br>-do these assumptions and costs vary according to the settings and circumstances?<br>-what is the scope of this variability?<br>-to what extent, and under what circumstances, can budgets and protocols be amended in light of stakeholder insights or interests?<br>-what are the key impediments to greater budget and protocol flexibility?
Planning	
**Goals and Procedures of the Science Program**	-what processes and procedures are required to conduct the science program?<br>-what changes will these processes and procedures require in the host community (e.g., demand for local labor, access to private property)<br>-what are the potential implications of these changes?
**Scope of CSE Strategy**	-under what circumstances is CSE necessary?<br>-who determines the need for CSE and its scope?<br>-how are these assessments made?<br>-how can CSE strategies be scaled to fit the scope of the science program?
**Identity of the Science Program**	-how do program team members identify themselves to stakeholders and host community members?<br>-how do stakeholders and host community members understand the identity of the science program?
**Site Selection**	-what features/capacities of the host community are necessary for effective CSE?<br>-how are these capacities affected by social, cultural, economic and political circumstances?<br>-how are site selection decisions made for science programs?<br>-how, and to what extent, are host community stakeholders involved in site selection decisions?
**Forecasting Relevant Interests and Stakeholders**	-what types of interests are likely to be affected by the program (e.g., regulatory, labor market, privacy, cultural identity)<br>-what individuals/organizations are most likely to hold/represent these interests (i.e., stakeholders)?<br>-what types of formative research and forecasting analyses are conducted for science programs?<br>-how do the results of these studies/analyses inform the design and management of the CSE strategies?<br>-what criteria and processes are used to identify and contact stakeholders?<br>-what forms of agreement or authorization (formal/informal) are necessary for the program to proceed?<br>-what are the terms of these agreements? Are the terms considered fair by all parties?<br>-what obligations and accountabilities do they create for the program/stakeholders?
**Termination of the Science Program**	-what plans have been made for winding down or terminating the science program?<br>-what are the implications of these plans for the CSE strategy?<br>-does the CSE strategy include explicit plans for the termination of the program, either on schedule or for unforeseen reasons?<br>-if so, what activities/processes are included in these plans?
Design	
**Design Constraints and Decisions**	-what design constraints for the science program/CSE strategy are identified in the CSE planning process (e.g., concerns re. labor market pressures, legal or regulatory constraints)<br>-how are these addressed?<br>-what are the budget implications?<br>-what are the implications for CSE? For the performance of the program?
**Listening and Deliberation**	-have specific opportunities for listening to stakeholders (individually or in groups) been built into the CSE strategy design?

	-have specific opportunities and contexts for deliberation with stakeholders been built into the CSE strategy design?<br>-under what circumstances is deliberation required, or invoked by stakeholders or the program team?<br>-what issues are subject to deliberation?<br>-have specific terms and procedures been designed to facilitate fair deliberation?<br>-what are the outcomes of these deliberations, e.g., do they result in changes or amendments to the program?
**Integration with Program Management**	-has the CSE strategy been designed to integrate effectively with the day to day management of the program, i.e., is there a pathway (e.g., senior leadership meetings) for reviewing insights arising from on-going interactions/relationships with stakeholders?
Management	
**Leadership**	-what types of leadership are required for the effective execution of a CSE strategy? See reference (14) for details
**Human Resources**	-what skills and abilities are sought in CSE team members (in cases where a CSE team is required)?<br>-what criteria are established to identify and hire the CSE team?<br>-what performance standards are expected of the CSE team (e.g., respectfulness, effective communications, enthusiasm) and how are these established?<br>-what training, supervision, and performance management are provided for the CSE team?
**Establishing and managing relationships with stakeholders**	-how are initial contacts and relationships established with stakeholders?<br>-how are relationships with stakeholders managed?<br>-how do stakeholders prefer to be engaged?<br>-how is the quality of stakeholder relationships monitored throughout the course of the program?<br>-what recourse do stakeholders have if they are unsatisfied with their relationship with the program?
**Communications**	-what information about the scientific program is communicated to stakeholders?<br>-how is the content and mode of communication determined?<br>-how is the utility and sufficiency of the information for stakeholders determined?
**Establishing a presence in the host community**	-what approaches are taken by the program to establish a presence and identify for the program in the host community (e.g., branding, educational or entertainment programing, retaining office space, strategic collaborations and associations)?<br>-what are the costs and implications of these activities for the program?<br>-what expectations do these approaches create among host community members and stakeholders? And how are these expectations determined and managed?
Evaluation	
**Process evaluation**	-what mechanisms of action and causal pathways can be described for the components of the CSE strategy and their related interactions, outcomes or implications?<br>-Do these descriptions, or their interpretation, vary with different perspectives (e.g., program team vs. various stakeholders)?<br>-what are the implications or consequences of such variability for assessments of quality or effectiveness?
**Outcomes and implications**	-what stakeholder interests have been identified by the CSE strategy?<br>-have stakeholders' interests been acknowledged and addressed to their satisfaction?<br>-for those interests that were not addressed to the satisfaction of the stakeholders, were there specific constraints or obstacles or rationales or commitments that prevented the program from addressing these interests?<br>-what are the consequences (positive and negative) of addressing stakeholder interests for the quality of the relationships with the relevant stakeholders, and for the performance of the program?<br>-does the program team believe they have taken stakeholders' interests seriously and treated them fairly? Do different team members have different views?<br>-do stakeholders believe their interests have been taken seriously and treated fairly by the program? Do different stakeholders have different views?<br>-how can these different perspectives be aggregated, or reconciled?<br>-have stakeholders' attitudes to the science program changed over the course of the program?<br>-how do stakeholders assess the trustworthiness of the program? Do these assessments change over time?<br>-what are the determinants of trustworthiness from stakeholder/program perspectives?<br>-have the benefits for the host community anticipated by the program been realized?<br>-if so, did the CSE strategy contribute to the realization of the benefits?<br>-if not, did CSE play a role?<br>-has the science program maintained good standing in the community? How has this been determined?<br>-has the program honored its initial ethical commitments to stakeholders and the host community?<br>-is further research planned for the site?
